# Metagenomic profiling of nasopharyngeal samples from adults with acute respiratory infection

**DOI:** 10.1098/rsos.240108

**Published:** 2024-07-10

**Authors:** Nurlan Sandybayev, Vyacheslav Beloussov, Vitaliy Strochkov, Maxim Solomadin, Joanna Granica, Sergey Yegorov

**Affiliations:** ^1^ Kazakhstan-Japan Innovation Centre, Kazakh National Agrarian Research University (KazNARU), Almaty, Kazakhstan; ^2^ TreeGene Molecular Genetics Laboratory, Almaty, Kazakhstan; ^3^ School of Pharmacy, Karaganda Medical University, Karaganda, Kazakhstan; ^4^ Michael G. DeGroote Institute for Infectious Disease Research, McMaster Immunology Research Centre; Department of Biochemistry and Biomedical Sciences, McMaster University, Hamilton, Ontario, Canada; ^5^ Department of Biology, School of Sciences and Humanities, Nazarbayev University, Astana, Kazakhstan

**Keywords:** acute respiratory infection, metagenomics, virome, nasopharyngeal swab

## Abstract

Diagnosis of acute respiratory infections (ARIs) is challenging due to the broad diversity of potential microbial causes. We used metagenomic next-generation sequencing (mNGS) to analyze the nasopharyngeal virome of ARI patients, who had undergone testing with a clinical multiplex PCR panel (Amplisens ARVI-screen-FRT). We collected nasopharyngeal swabs from 49 outpatient adults, 32 of whom had ARI symptoms and were PCR-positive, and 4 asymptomatic controls in Kazakhstan during Spring 2021. We assessed the biodiversity of the mNGS-derived virome and concordance with PCR results. PCR identified common ARI viruses in 65% of the symptomatic cases. mNGS revealed viral taxa consisting of human, non-human eukaryotic and bacteriophage groups, comprising 15, 11 and 28 genera, respectively. Notable ARI-associated human viruses included rhinovirus (16.3%), betaherpesvirus 7 (14.3%) and Epstein-Barr virus (8.16%). The primary phage hosts were *Streptococcus* spp. (32.7%), *Pseudomonas aeruginosa* (24.5%) and *Burkholderia* spp. (20.4%). In total, 47% of ARIs were linked solely to bacterial pathogens, a third to viral-bacterial co-infections, and less than 10% to only viral infections by mNGS. PCR showed low concordance with mNGS, except for rhinovirus. These results underscore the importance of broad diagnostic methods and question the effectiveness of commonly used PCR panels in ARI diagnosis.

## 1. Introduction

Acute respiratory infections (ARI) include conditions such as the common cold, bronchitis, pharyngitis and rhinosinusitis. ARI are the leading cause of outpatient visits and antibiotic prescriptions globally [1,2]. Most ARI are considered viral, although bacterial ARI are not uncommon and can develop after viral infection [3–5]. Clinical management of uncomplicated ARI is symptomatic or empiric, especially in the limited-resource settings of developing countries [2,5,6]. Laboratory testing of ARI, typically indicated when a bacterial infection is suspected, is done using microbiologic and molecular assays targeting ‘common’ respiratory pathogens [7]. Often, the underlying pathogens remain untyped in over a third of ARI cases, and it is unclear whether the pathogens identified by targeted assays are the true cause of ARI symptoms [3,8,9].

These diagnostic gaps have stimulated studies of the respiratory virome, including not only well-known pathogenic viruses but also less-characterized viral communities that may play a role in health and disease [10,11]. The nasopharyngeal virome, in particular, is a complex and dynamic ecosystem represented by human, non-human eukaryotic host and bacteriophage sub-groups [10,12]. The metagenomic characterization of the respiratory virome holds promise both in the context of small-scale clinical settings, where it could facilitate the diagnosis of respiratory conditions [13,14], and in the context of larger scale public health surveillance, where it could provide insights into the population-level dynamics of pathogen circulation [15,16], informing the clinical guidelines [17].

Metagenomic next-generation sequencing (mNGS) is a powerful tool to explore the respiratory virome, offering an unbiased approach to detect known and novel viruses [10]. However, the concordance between mNGS and traditional diagnostic methods, like multiplex PCR, varies across different platforms and for different pathogens [8]. The contribution of bacterial-viral co-infections and the role of ‘uncommon’ viruses and bacteria in ARI patients from diverse demographic and geographical strata are also not well understood.

In our earlier work, we surveyed adult ARI outpatients in Kazakhstan during a period of low COVID-19 transmission in Spring 2021 [18,19]. One global hallmark of this period was absent/low transmission of influenza and respiratory syncitial virus (RSV), but a high incidence of human rhinovirus (HRV) [20,21]. Here, we further characterized the nasopharyngeal virome associated with ARI using mNGS as part of the national initiative to enhance the diagnostic capacity for ARI in the post-pandemic era [22]. Our specific endpoint was to compare the mNGS results to those of an ARI-targeting PCR panel used commonly by clinical laboratories in Kazakhstan and the neighbouring countries [23–25].

## 2. Materials and methods

### 2.1. Study setting

This study is a follow-up to our earlier work exploring virologic causes of ARI among outpatients of two public hospitals in Kazakhstan [18,19]. In this earlier study, 50 participants with ARI symptoms, who tested negative for SARS-CoV-2 and influenza A virus, were recruited between May 18 and June 7, 2021. The highlights of this period in Kazakhstan were the deployment of mass COVID-19 vaccination [26–28], low SARS-CoV-2 infection rates but a concomitant increase in non-COVID respiratory infections [18].

Written consent to participate was obtained from all participants and witnessed by the study coordinator. ARI was defined by the presence of the following respiratory symptoms: fever, nasal congestion with/without rhinorrhoea, cough, sore throat and lymphadenopathy. Nasopharyngeal swabs (NPS) were collected and partitioned for PCR and mNGS assays as described earlier [18]. In addition to the ARI samples, we collected NPS from four asymptomatic controls (AC). Due to limited sample availability, we excluded one ARI sample from the mNGS assays; this resulted in a total of 53 samples (49 ARI and 4 AC) processed and analysed in the current study.

### 2.2. mNGS

Initially, to eliminate intact host cells and ensure that the extracted nucleic acids would be mainly of viral origin, NPS were enriched using a combination of low-speed centrifugation and filtration following virome characterization protocols [18,29,30]. Specifically, NPS were centrifuged at 5 200 g for 10 min, followed by centrifugal filtration at 13 000 g for 1 min using 0.45 µm cellulose acetate filters (Spin-X Centrifuge tube filter, Corning). Free nucleic acids were removed using Pierce Universal Nuclease (Thermo Fisher Scientific). Samples were concentrated using a Pierce ™ PES protein concentrator (Thermo Fisher Scientific) at the 100 K molecular weight cutoff.

Sequencing library preparation was done as described earlier [18]. Briefly, both genomic DNA and RNA were extracted using the MagMAX Total Nucleic Acid Isolation Kit (Thermo Fisher Scientific), followed by cDNA synthesis, amplification, and primer removal using the SeqPlex RNA Amplification Kit (Sigma) yielding 150–400 nucleotide cDNA fragments. The resulting cDNA quality/purity and quantity were assessed by NanoDrop and Qubit, respectively.

Libraries were prepared using the Ion Plus Fragment Library Kit, Ion Xpress Barcode Adapters 1–96 Kit (both from Thermo Fisher Scientific), and the Agencourt AMPure XP kit (Beckman Coulter). Barcoded libraries were assembled using the Ion Plus Fragment Library Kit and Ion Xpress Barcode Adapters 1–96 Kit and quantified according to the Ion Library TaqMan Quantitation Kit's protocol. Libraries were then loaded onto Ion 530 Chips with the Ion Chef Instrument and processed on the Ion Torrent S5 System (Thermo Fisher Scientific).

### 2.3. Multiplex PCR panel

Real-time PCR was performed on the NPS genomic DNA and RNA using the Amplisens ARVI-screen-FRT kit (Amplisens, Interlabservis, Moscow, Russia). The PCR panel targeted both RNA (respiratory syncytial virus (RSV), metapneumovirus (MPV), human parainfluenza virus-1–4 (HPIV), coronaviruses (HCoV) ОС43/HKU-1 and NL-63/229E and rhinovirus (HRV), and DNA (adenovirus (Adv) B, C and E and bocavirus (BoV) viruses [18].

### 2.4. Bioinformatic analyses

Sequencing reads were assigned taxonomic information using the open-source Chan Zuckerberg ID (CZID) portal (v7.0, analysis performed in June 2022), which compares queries assembled sequences against the NCBI nucleotide and protein databases [31]. In addition, CZID v7.0 incorporates adapter trimming, low-quality read removal, low complexity read removal, external RNA controls consortium read and human genome (GRCh38) read removal [31].

Independent of the CZID analysis, we also implemented EDGE [32] to process the reads. We then used a combination of read-based taxonomic classification tools, including Genomic Origin Through Taxonomic CHAllenge (GOTTCHA2) [33], Kraken2 [34], Burrows-Wheeler Alignment tool [35], MethaPhlAn2 [36] and Diamond [37] to compare and validate the taxonomic readouts with the CZID-generated results.

The data were standardized based on unique reads mapped per million input reads at the genus tier. To eliminate background and low-frequency sequencing reads, we followed guidelines from the recent metagenomic studies [38,39] and set taxon inclusion criteria as follows: nucleotide reads per million (NT %id) of 95% or higher, the alignment length (NT L) of ≥70 base pairs, and reads per million (rpM) > 1. The recovered bacteriophage genera were grouped based on their respective bacterial hosts using the NCBI taxonomy browser (https://www.ncbi.nlm.nih.gov/Taxonomy/Browser/wwwtax.cgi).

### 2.5. Statistical analyses

Taxon abundance heatmaps were constructed using log-transformed normalized rpM and graphed using Morpheus (https://software.broadinstitute.org/morpheus/). Simpson's biodiversity indices were calculated on the normalized rpM data via BiodiversityR [40], using the default recommended settings, and plotted using R ggpubr [41]. Differences across the PCR and AC sub-groups were assessed using the Mann-Whitney U and Kruskal-Wallis tests. Association rule mining was performed in Orange 3.36.2 [42]. The diagnostic performance of the Amplisens PCR panel was assessed by calculating the sensitivity and specificity metrics using mNGS as the reference standard in R v. 4.3.2 (R Foundation for Statistical Computing, Vienna, Austria). Sensitivity was calculated as the proportion of positive cases identified by both PCR and mNGS (true positives) divided by the total number of cases confirmed by mNGS. Specificity was determined by dividing the number of participants who tested negative by both PCR and mNGS (true negatives) by the total number of participants who were negative for the PCR panel pathogens according to mNGS.

## 3. Results

### 3.1. Nasopharyngeal virome characteristics

The median age of ARI participants was 32 years and 73.5% of them were female; the median age of AC participants was 36 years and 50% of them were female. In 65% (32/49) of ARI participants, multiplex PCR was positive for HPIV-3 (1/49), HPIV-4 (24/49), HRV (2/49), HPIV4-HRV (4/49), HAdV (1/49) or HCoV (1/49). mNGS was performed on both ARI (*n* = 49) and AC (*n* = 4) samples, with a median of 406,954 reads obtained per sample after filtering out human reads. The initial validation and comparison of taxonomic outputs generated by the different pipelines indicated presence of similar viral taxon signatures across the different taxonomy pipelines. Therefore, we used the CZID analysis output for the rest of the analysis.

**Figure 1.**
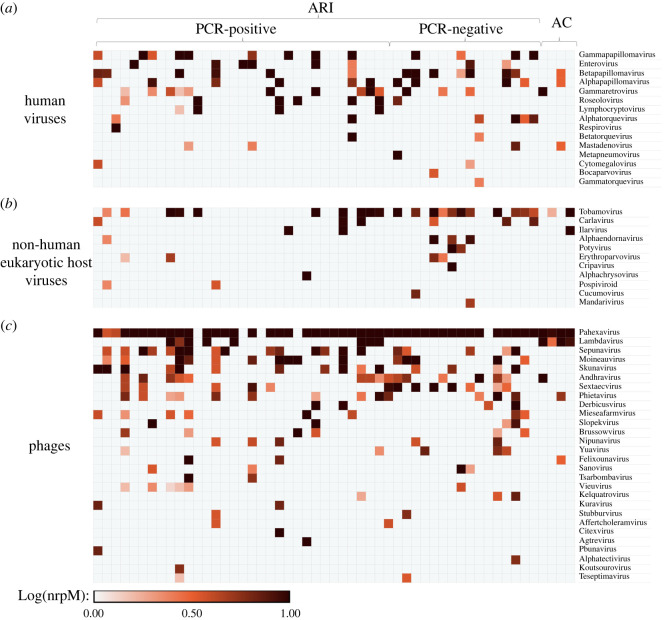
Nasopharyngeal virome profile of participants with acute respiratory symptoms (ARI), stratified by the multiplex PCR result, and asymptomatic controls (AC). Each row is a viral genus. Each column represents a participant. Microorganism abundance was derived by log-transformation of normalized reads per million (nrpM). Viral taxa were plotted in order of descending mNGS-derived cumulative abundance across the cohort. (*a*) Human viruses. (*b*) Non-human eukaryotic viruses. (*c*) Phages.

After bioinformatic processing, the viral taxa were stratified into human, non-human eukaryotic host, and bacteriophage sub-groups (figure 1), including 15, 11 and 28 genera, respectively (electronic supplementary material, tables S1 and S2). Among the human viruses, gammapapillomavirus and enterovirus were most prevalent in symptomatic participants and observed in 13/49 (26.5%) and 8/49 (16.3%) participants, respectively. No HPIV-4, the virus most prevalently detected by PCR, was detectable by mNGS. Non-human eukaryotic host viruses consisted predominantly of plant and fungal viruses, of which Tobamovirus, derived from tobacco and other Solanaceae, was most prevalent among all participants (in 23/53, 43.4%). Among the bacteriophages, Pahexavirus, a *Propionibacterium (Cutibacterium) acnes* bacteriophage, was most prevalent and abundant, observed in 48/53 (90.6%) participants and in both ARI and AC groups (electronic supplementary material, table S3).

**Figure 2.**
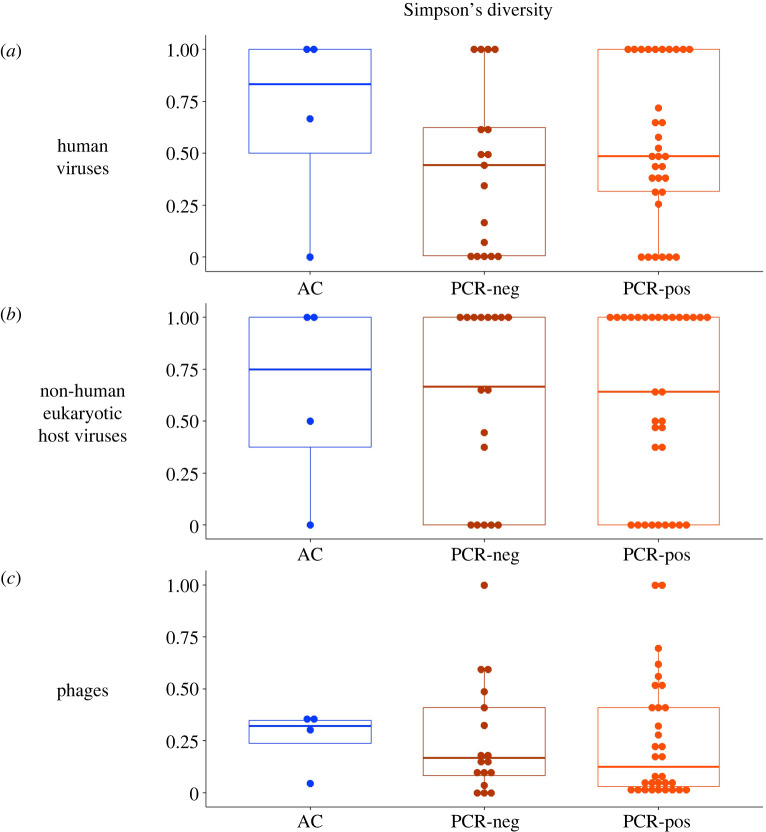
Simpson's alpha biodiversity indices for each of the three major virus sub-groups: (*a*) Human viruses, (*b*) Non-human eukaryotic viruses and (*c*) Phages in the nasopharynx of asymptomatic controls (AC) and subjects with acute respiratory infection either with negative (PCR-neg) or positive (PCR-pos) test results on a multiplex PCR targeting common respiratory viruses (see Methods for the PCR panel description). Each box plot displays a median value for the diversity index (thick lines) and interquartile ranges (boxes).

Further, we hypothesized that the nasopharyngeal virome diversity would differ between the participant sub-groups stratified by the multiplex PCR test result. To test this, we compared Simpson's alpha-diversity index across the PCR-positive, PCR-negative and AC groups (figure 2). No significant differences were observed in any of the comparisons (figure 2).

### 3.2. Distribution of ARI-linked viruses and bacteria

**Figure 3.**
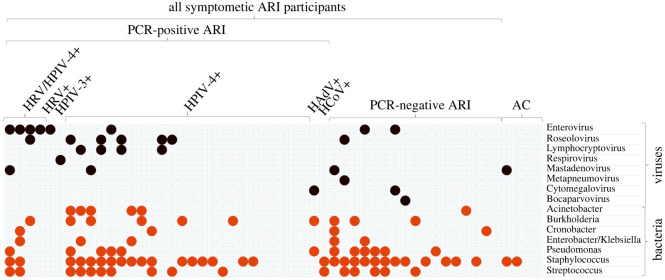
Nasopharyngeal profile of ARI patients, consisting of human viruses and bacteria known to cause respiratory disease. Each row is a viral (brown) or bacterial (orange) genus. Columns represent participants grouped by their ARI/AC status and by multiplex PCR results. PCR-positive samples are further stratified by the PCR-identified virus. Metagenomically detected microorganisms are shown as present (coloured circles) or absent (no circles). Viral taxa were plotted in order of descending mNGS-derived cumulative abundance across the cohort (similar to figure 1). Bacterial genera were derived from the list of mNGS-recovered bacteriophages using phage-host classification. ARI: acute respiratory infection. AC: asymptomatic controls. HRV: human rhinovirus. HPIV: human parainfluenza. HAdV: human adenovirus. HCoV: human coronaviruses (seasonal).

We then focused on pathogens known for their associations with respiratory disease, narrowing our analysis to 8 human virus genera and 7 bacterial taxa representing hosts of the 16 recovered bacteriophage genera (figure 3). Here, we observed a co-detection of viral and bacterial pathogens in 16/49 (32.7%) ARI cases. Only a bacterial or viral pathogen was present in 23/49 (46.9%) and 4/49 (8.2%) ARI cases, respectively. No viral or bacterial pathogens were seen in 6/49 (12.2%) ARI cases (figure 3). The top three ARI-associated human viruses were enterovirus (16.3%, human rhinovirus, HRV-A), roseolovirus (14.3%, human betaherpesvirus 7, HBV-7) and lymphocryptovirus (8.16%, Epstein-Barr virus, EBV) (figure 3). The top three ARI-associated phage hosts were *Streptococcus* spp (32.7%), *Pseudomonas aeruginosa* (24.5%) and *Burkholderia* spp. (20.4%). The virome of both asymptomatic and symptomatic subjects was also abundant in the *Staphylococcus* (60.4%) bacteriophages. Except for mastadenovirus and *Staphylococcus*, all other respiratory pathogens were observed only in ARI but not in AC.

HRV was detected by mNGS in all PCR + HRV cases, in addition to two PCR-negative cases and one subject with a PCR-identified HPIV-4 infection. HBV-7 was detected as a viral mono-infection in two cases, and as a co-infection in 5 cases with HRV (*n* = 1), EBV (*n* = 3) and MPV (*n* = 1). EBV was detected in a total of 4 samples, of which only one was a viral monoinfection. We then further explored co-infection patterns using association rule mining to describe frequently co-occurring pathogens using ‘rules’ predicting the presence of a pathogen based on the occurrence of other pathogens. Using a minimal support threshold of 6% (occurrence in >=3 participants), two associations were identified. Specifically, *Staphylococcus* spp. co-occurred with *Enterobacter/Klebsiella*, and with Mastadenovirus in 9.4% (leverage = 3.7%) and 7.5% (leverage = 3.0%) of the cases, respectively (lift = 1.66 for both, indicating strong associations). The observed positive leverage indices indicated that the pathogens co-occurred more often than would be expected based on their individual frequencies alone.

### 3.3. Concordance between mNGS and Amplisens PCR

The PCR and mNGS results were relatively concordant for human rhinovirus (HRV) with PCR sensitivity and specificity relative to mNGS of 62.5 (95% CI 24.5, 91.5) and 100% (95% CI 92.1, 100), respectively. The concordance between mNGS and PCR for other PCR panel targets, including human parainfluenza (HPIV), adenovirus (HAdV), bocavirus (BoV) and seasonal coronavirus (HCoV) was low (electronic supplementary material, table S4).

## 4. Discussion

Here we characterized the nasopharyngeal metagenome associated with ARI and assessed the concordance between mNGS and the Amplisens PCR widely used by the laboratories in the region [23–25]. In the absence of influenza and RSV [18], our findings were consistent with other recent studies, where viral and bacterial ARI were predominantly associated with rhinovirus and *Streptococcus* spp., respectively [3].

We observed a relatively high concordance between PCR and mNGS for HRV but low concordance for other PCR targets, such as HPIV and HAdV. To the best of our knowledge, this is the first published assessment of the Amplisens ARVI-screen-FRT PCR performance using mNGS, warranting further investigation of the clinical validity and value of using this commercial assay for ARI diagnosis. The discrepancies observed between mNGS and PCR may be attributed to the known differences between the two assays in sensitivity and specificity [8,13,14,16]. The complete lack of HPIV-4 (human orthorubulavirus 4) in the mNGS output could be due to very low nasopharyngeal HPIV-4 loads or a non-specific detection by PCR. Interestingly, our mNGS data also demonstrated a large fraction of the ARI (47%) to be associated with only respiratory bacteria— consistent with bacterial sinusitis or tonsillitis—but not viruses. Moreover, half of the mNGS-identified ‘bacterial-only’ samples were also HPIV-4 positive by PCR. This suggests that bacterial/bacteriophage load may be overwhelming and/or interfering with the molecular signal of HPIV-4 infection.

Remarkably, 33% of the ARI in our study were associated with co-detected viruses and bacteria, while viral mono-infections contributed to just a small ARI fraction (8%). These findings are in line with negative virus-to-virus interactions and positive interactions seen between viral and bacterial pathogens [3,43–45]. The co-occurrence we observed for *Staphylococcus* and *Enterobacter/Klebsiella* is consistent with a report of co-colonization by these pathogens of the respiratory tract in intensive care unit patients [46].

To the best of our knowledge, the almost ubiquitous presence in NPS samples of Pahexivirus, a *Propionibacterium acnes* bacteriophage, has not been previously reported. However, a recent thesis [47] described a lower relative nasopharyngeal abundance of both Pahexivirus and *Propionibacterium acnes* in HIV + subjects compared to HIV-negative controls from Malawi, which may reflect a commensal nature and association of *Propionibacterium acnes* with the systemic immune state.

The identification of lymphocryptovirus (EBV), a common cause of infectious mononucleosis, and roseolovirus (HBV-7) as top ARI-associated human viruses warrants public health consideration. Since HBV-7 primarily causes roseola infantum or sixth disease in young children, typically manifesting with a high fever and a skin rash [48], our data suggest that adults could be important HBV-7 reservoirs within communities.

Our findings should be interpreted in the light of the limitations. First, the relatively small sample size affected our ability to discern complex patterns of viral and bacterial (co-)infection patterns. The low concordance between mNGS and PCR for certain viral targets, such as HPIV and HAdV, could be due to viral variants but we were unable to address this. One potential means to increase the odds of detection by mNGS of low abundant viruses would be to sequence more reads per sample—something that we could not do due to limited study funds. The large fraction of ARIs potentially associated only with respiratory bacteria, particularly in HPIV-4+ PCR samples, suggests that further optimization of our mNGS methodology is warranted prior to deployment in clinical settings. We could not validate the identified bacterial species as other assays, such as serology, antigen detection or complement fixation, were unavailable during the study. Lastly, our analysis pipeline did not account for the presence of fungal pathogens, which could contribute to ARI pathogenesis.

Despite the limitations, our study highlights the multifaceted nature of the nasopharyngeal virome in ARI, particularly adding to other studies showing the feasibility of deploying mNGS in the limited-resource settings of developing countries [15,49]. However, the discrepancies between mNGS and multiplex PCR raise questions about the value of the Amplisens PCR panel in the clinical management of ARI and punctuate the need for the integration of complementary clinical and laboratory methods to enhance detection accuracy of ARI pathogens [50].

## Data Availability

The raw mNGS data have been deposited to NCBI under accession PRJNA904925 (https://www.ncbi.nlm.nih.gov/bioproject/?term=PRJNA904925). The data are provided in electronic supplementary material [51].
